# Foreign Summary

**Published:** 1839-03-09

**Authors:** 


### FOREIGN SUMMARY.

*Case of Hepatic Abscess Opening into the Stomach by three perforations, also into the Pericardium;—Pericarditis;—Pleuritis. With Remarks.* By Professor Graves.—The following case contains many particulars of extreme interest, among which I beg to direct the reader's attention more especially to the physical phenomena produced by the simultaneous presence of air and fluid in the pericardial sac, no instance having been hitherto recorded where similar symptoms, arising from ulceration extended to that sac, have been observed.

In order not to lengthen the case too much, I have omitted the details of treatment; they consisted of local depletion in the first instance by means of leeches, and an attempt to mercurialize the system, which attempt failed, because suppuration was in all probability established before it was made. My experience confirms the assertion made by Annesley and other writers on diseases of tropical climates, that it is impossible, or at least very difficult, to make the mouth sore to salivation, once the formation of abscess in the liver commences. Of course, no practitioner who is aware that hepatic suppuration has actually set in, will continue the exhibition of mercury; it then becomes injurious. In the following case, when suppuration was ascertained, poultices were applied, and various astringents were subsequently employed, in vain, to check the diarrhœa.

Anne Walker, æt. 25, spinster, of spare habit and nervous temperament, on Thursday night, 13th inst., without any assignable cause, was seized with a sudden and violent pain in every part of the abdomen, extending to the loins and back, unpreceded and unaccompanied by any other complaint: was immediately bled, but without much relief; continuing in the same state, venesection was repeated the next morning with more effect; hot stupes were also applied. The entire of the 14th (yesterday) she remained in excruciating agony, applying the stupes, and obtained but little ease. She now lies on the back, with the legs drawn up towards the body, unable to turn to either side, or stir in the least in the bed, without an insupportable increase in her complaints; the pains she describes as of a lancinating nature, sometimes resembling the pricking of a number of pins, commencing at the epigastrium, shooting downwards to the pubes, and extending laterally into each hypochondriac and lumbar region.

Since the commencement of the attack, she has been deprived of sleep; much annoyed with constant thirst, and a nauseous, disagreeable taste in the mouth. Her countenance is now anxious and distressed; skin moist, and covered with slight perspiration; tongue, white and moist; pulse 128, small, and somewhat wiry; respiration 54; no morbid phenomenon can be detected in the chest; heart's action rapid, and sounds natural; the abdomen is tense, hard, and exquisitely painful, the slightest degree of pressure causing much uneasiness; bowels free; urine passed in regular quantities.

17th. The greater part of the night was in a profuse perspiration; the pains in the abdomen generally not so acute, they are, however, still aggravated by change of position: the mouth has become tender, and gums spongy; pulse 104, tolerably full, and easily compressed; respiration 40; tongue coated and moist.

18th. Since the poultices were applied, the pains have been so far lessened that she can extend her legs without their being increased; her countenance is not so distressed, and she appears more at ease; is at present in a profuse sweat; pressure on the abdomen still occasions uneasiness. In the right hypochondrium and epigastrium there is a considerable tumefaction, somewhat of a conical shape, affording, when pressed, a degree of elasticity and *dulness on percussion;* the pain produced in this part by pressure is very acute, whilst elsewhere it is comparatively slight.

19th. The only part of the abdomen pained by pressure is that where the tumefaction was observed yesterday; it extends from below the ensiform cartilage to within a couple of inches of the umbilicus, also laterally occupying a space between three and four inches; and to-day a sensation of fluctuation is communicated to the touch.

20th. A violent purging commenced yesterday, and continued the entire night; stools numerous, eight or ten, liquid, and of a dark colour, each being attended with griping and kneading; was much troubled with shiverings and pains in the back; her breathing is more distressed, and accelerated, 44 in the minute; pulse 132, small and hard; tongue moist. No change has taken place in the appearances of the abdomen.

24th. There has been no return of the purging since the 21st; the perspirations are diminished, and her general aspect is improved; she now complains principally of pains in the back, continued, and shooting upwards along the entire of the spinal column. When the tumour is now percussed, *it emits a tympanitic resonance;* the lower part of the left side also is very clear on percussion; *cannot now detect the fluctuation observable on the 19th;* the elasticity remains as before; pulse 116, soft and improved in strength; respiration 30.

26th. Was troubled with a hiccough the entire night; had but little sleep, and sweated profusely; is quite free from pain except in back and loins; has no appetite, but great desire for drinks; the tumour appears flatter, is free from tenderness, and still tympanitic when percussed; pulse 128, small and soft, respiration 32; breathing regular. Tube of stomach pump to be passed into œsophagus.

28th. No air escaped after the tube was introduced; no change has taken place either in the size or sound of the tumour; bowels freed three times since yesterday, and stools attended with griping.

29th. The tumour in epigastrium is considerable diminished in size, percussion elicits, as before, a tympanitic resonance, but does not extend, as on previous days, to the right hypochondrium; her countenance is improved, and spirits not so depressed; breathing continues too free, and pulse rapid.

Oct. 1st. Purging has returned, with griping pains in the abdomen, and numerous liquid stools; the tumour in abdomen is scarcely perceptible, and but a slight degree of clearness on percussion can be elicited; the upper part of the tongue is extremely painful; on the dorsum there are two or three sores, the largest about the size of a silver penny; the others resemble fissures, and are separated from each other by septa: pulse 116, soft and tolerably full; respiration 32.

2d. Purging remains unchecked; the tumour in abdomen has altogether disappeared; no tympanitic resonance is now afforded by percussion; the sides of the tongue this morning are covered with aphthæ; the sores on the dorsum remain the same.

3d. No effect has been produced on the purging, was upwards of six times to stool since yesterday; is much reduced in strength; countenance pale; pulse quick, 112; has great thirst; tongue dry, and not so sore.

6th. Heart's sounds natural. Percussion and respiration over both lungs as in the healthy state: abdomen sunken and free from pain.

7th. Bowels have been opened seven times within the last twelve hours. Pulse 120. Respiration 30.

9th. *Was attacked yesterday with acute pain in the cardiac region, and last night had a violent beating of the heart, also a burning heat below the left breast.* She cannot recollect any cause to which she might attribute this. Her present state is extreme emaciation and debility, cheeks hollow, eyes sunken, countenance dejected, and spirits languid; her breathing remains accelerated, short, and distressed; the jugular veins in the recumbent posture turgid, but without pulsation; likewise those along the trachea.

Percussion over chest generally is clear, except at the inferior and middle portions of the left side. Respiration in these parts is feeble, elsewhere pure and loud: impulse of heart perceptible, but feeble. About half an inch distant from the lower edge of the mamma both sounds are confused, and a slight bruit de soufflet is audible; advancing to the right it increases in roughness, and below the mamma it becomes a complete creaking noise, accompanying both sounds of the heart, and is still louder between the sternum and breast; when pressure is applied it gradually increases these phenomena, and when considerable pressure is used, they are changed into a loud frottement, obscuring both sounds, the first especially; they are also rendered more distinct by holding the breath.

Abdomen smaller; purging stopped; pulse 130, small and compressible.

10th. The phenomena are now audible as far at the middle of the sternum, over the cardiac region, and laterally, being in each place of the same character. The sound is between bruit de soufflet and bruit de scie, in a great measure masking the first sound and accompanying the second, which still retains its clearness. Immediately under the mamma, together with these sounds, but heard only occasionally, *is a peculiar metallic click*, affording the idea of some fluid dropping in or about the pericardium; it is removed when pressure is made over the heart, whilst the other noises undergo a thorough change; thirst urgent.

11th. Has not had a return of the pains in the left side; sweats every night as much as hitherto: had several shiverings last night, after each of which she fell into a copious perspiration. Pulse 136, feeble; respiration 40; bowels regular.

Impulse of heart is feebler; when the hand is placed over it a rubbing sensation is communicated.

*The sound to-day has assumed the character of an emphysematous crackling, is very fine, and obscures both sounds of the heart;* is more distinct along the middle and inferior parts of the sternum, and can also be heard to the left of the mamma. *The metallic click, or apparently the dropping of fluid, observed yesterday, is more audible and distinct, but irregular in frequency.*

12th. The irregular click, audible yesterday only at intervals, has now become *a loud metallic ticking, audible at each stroke of the heart* over those parts where the emphysematous crackling and other sounds were to be heard; it obscures all the phenomena hitherto noted, except a slight bruit de soufflet about the nipple of the left mamma. Impulse cannot be felt. Is sinking fast.

13th. Died last night at 10 o'clock.

*Autopsy twelve hours after Death.*—Percussion over the front of chest afforded no evident dulness; over the cardiac region it was clear. When the sternum was raised, both lungs were found collapsed; the left in particular, which was found compressed by a quart of sero-purulent fluid. Weak adhesions connected both lungs with the external pericardium; and their inferior lobes with the upper surface of the diaphragm. The pericardium appeared enlarged, and a small quantity of fluid could be felt.

The abdominal parietes being removed, the cavity of a large abcess was exposed, situated in the left lobe of the liver. Its form was circular, about eight inches in circumference, and bounded anteriorly by a portion of the parietes of the abdomen, and ensiform cartilage. Its posterior wall was formed by the remaining solid part of the left lobe; whilst the diaphragm superiorly was in immediate connexion with it, and the falciform ligament served as a means of separation between it and the right lobe: its thin edge was over-lapped by a portion of the stomach; and near the pyloric orifice was an ulcerated circular hole, with rounded and smooth edges, about three-quarters of an inch in diameter, communicating directly with the abscess. The stomach was intimately connected with the sub-surface of the left lobe by its concave margin; and near to its cardiac extremity were two other openings, one somewhat oval in shape, about half an inch in diameter, and connected with the abscess by means of a canal capable of admitting the tip of the little finger, and separated from the other by a thick band, evidently a portion of the stomach. This last perforation, or the one nearest the œsophageal extremity of the stomach, had no communication with the abscess. The surface of the abscess is irregular, presenting many depressions and elevations; its colour of a yellowish grey, its substance creamy, soft, and reduced by pressure into a pus-like fluid; when cut into, it is at least three-quarters of an inch in depth, but does not retain the same thickness in every part; beneath, the structure of the liver is visible, and in firm connexion with it the stratum of diseased substance, neither can it be separated from it.

Where the diaphragm and pericardium are united, *is a perforation sufficiently large to admit the middle or ring finger, and opening directly from the abscess into the pericardium*; the edges are ulcerated and uneven; and within the covering of the heart are about two ounces of yellow-coloured fluid mixed with flakes of lymph. The pericardial sac is increased to four times its natural thickness, but appears equally dense in all parts; its external surface is highly vascular; its interior is likewise inflamed, dotted with numerous red spots, in some parts about the size of a pin's head, and in others forming an arborescent appearance; the surface has in a great measure lost its natural glistening appearance, and looks uneven, being coated in parts with small portions of organized lymph; and generally, particularly towards the origins of the great vessels, with small, granular, semi-transparent bodies resembling millet seeds, or the eruption sometimes seen in cases of rheumatic fever: its feel is quite gritty, but when these bodies are scraped off, the serous lining of the pericardia is apparent underneath.

The heart itself is of a light red colour, and its investing membrane is covered, like the pericardial sac, with those granular substances, more abundant about the auricles and base of the heart. Both auricles are bound down to the substance of the heart, by means of strong, tough, and organized pieces of lymph.

Some tubercles scattered through the superior lobe of each lung. No adhesions existed between the peritoneum and intestines, or between these latter.

I am indebted to my talented and indefatigable clinical clerk, Mr. Thomas Moore, for the preceding report of the progress of this singular case, concerning which the following remarks appear necessary:

1st. When the abscess burst into the stomach, the epigastric tumour which the abscess formed, did not at once subside, but suddenly, from having yielded a dull sound on percussion, became tympanitic and clear; air from the stomach having found its way into the cavity, while the pus escaped.

2dly. The now tympanitic tumour seemed so exactly to resemble the stomach distended with air, that we were induced to pass a tube into the stomach, but it did not give vent to any air.

3dly. In a few days the air also passed from the cavity of the sac, then all traces of the tumour entirely and unaccountably disappeared.

4thly. The diarrhœa was caused by the perpetual flow of fetid and irritating matter from the abscess into the intestinal cavity.

5thly. No peculiar symptom, pain, or derangement of its functions, denoted the extensive ulceration of the stomach.

I shall revert to this subject after the details of the two following cases of ulceration of the stomach have been laid before the reader.

6thly. The inflammation spread by continuity of structure, from the abscess to the pleura and pericardium *in the first instance.*

7thly. Soon after the pericarditis thus formed had commenced, and at the time that its usual physical phenomena were clearly perceived, a new set of physical phenomena arose, *dating from the moment the pericardium was perforated, and air entered its sac.*

8thly. Although most intense general peritonitis existed when the patient was admitted, yet no traces of general peritoneal inflammation were discovered on dissection.

9thly. It may be asked, why I had not recourse to an operation to let out the matter, as soon as fluctuation had become plainly perceptible in the hepatic tumour? My answer is, that the tumour formed so quickly, and seemed to tend to the surface so rapidly, that I thought it better to wait for a day or two in order to render the operation safer, never anticipating that the matter could, in so short a time, find an exit by another channel.—*Dub. Jour. of Med. Scien. for Jan.*

---

*On the Use of Arsenic in some Affections of the Uterus.* By HENRY HUNT, Esq., of Dartmouth.—The first of these cases was that of a female of forty, who was much benefited by doses of from four to ten drops, three times a day, of the mineral solution, in a cancer of the uterus, which was in a state of ulceration, and was accompanied with much fœtid discharge, and great suffering.

Recollecting the inflammation of the pudenda, which occasionally attended the employment of this agent, the author thought it not unlikely that arsenic might have some favourable operation in other diseases of those parts, and he has accordingly employed it with advantage in cases of profuse and frequently occurring menstruation; in pain or tenderness of the uterus; and in neuralgia coming on regularly at the menstrual period, and supposed to be occasioned, therefore, by some irritation in the uterus. The arsenic is given in the form of pill, in doses of about one-twentieth of a grain, three times a day; and the author found, that in cases of great susceptibility to its action, the exhibition of the pill immediately after meals has been favourable.—*Edinburgh Medical and Surgical Journal.*

